# Bone Metastasis From Gastric Adenocarcinoma—What Are the Risk Factors and Associated Survival? A Large Comprehensive Population-Based Cohort Study

**DOI:** 10.3389/fonc.2022.743873

**Published:** 2022-03-25

**Authors:** Lei Huang, Yajie Zhao, Yan Shi, Weiguo Hu, Jun Zhang

**Affiliations:** ^1^Department of Oncology, Ruijin Hospital, Shanghai Jiao Tong University School of Medicine, Shanghai, China; ^2^Department of Geriatrics, Ruijin Hospital, Shanghai Jiao Tong University School of Medicine, Shanghai, China; ^3^Medical Center on Aging of Ruijin Hospital, Shanghai Jiao Tong University School of Medicine, Shanghai, China; ^4^State Key Laboratory of Oncogenes and Related Genes, Shanghai Jiao Tong University, Shanghai, China

**Keywords:** gastric adenocarcinoma, bone metastasis, long-term survival, cumulative incidence function, Fine–Gray subdistribution hazard regression, competing risk analysis, large population-based cohort study

## Abstract

**Background:**

While bone metastasis is not common in gastric adenocarcinoma (GaC), it can have important impacts on prognosis. This large cohort study aimed at exploring factors associated with bone metastasis in GaC and investigating the time-dependent cumulative mortalities and prognostic factors in GaC patients with bone metastasis at the population level.

**Methods:**

Data on patients with GaC diagnosed in 2010–2016 were retrieved from a large population-based database. We explored factors associated with bone metastasis using the multivariable-adjusted logistic model. We then calculated the time-dependent cancer-specific mortalities in GaC patients with bone metastasis using the cumulative incidence function and compared mortalities across subgroups using Gray’s test. We further assessed factors associated with mortality using the multivariable-adjusted Fine–Gray subdistribution hazard model.

**Results:**

Together 11,072 eligible patients with metastatic GaC were enrolled, which comprised 1,511 (14%) people with bone metastasis and 9,561 (86%) with other metastasis, encompassing 6,999 person-years of follow-up. Bone metastasis was more frequently detected in 2014 or later, in younger patients, in patients with gastric cardia cancers, in people with signet-ring cell carcinoma, and in those with poorly differentiated/undifferentiated cancers; it was less commonly observed in black patients. Bone metastasis was associated with more frequent brain and lung metastases. The median survival of patients with bone metastasis was 4 months; the 6-month and 3-year cancer-specific cumulative mortalities were 56% and 85%, respectively. In patients receiving chemotherapy, American Indians/Alaskan Natives, patients with gastric antrum/pylorus cancers, and those with positive lymph nodes had higher mortality risks, while those undergoing resection had lower mortality hazards.

**Conclusion:**

In GaC patients, bone metastasis was associated with various clinicopathologic factors including age, ethnicity, tumor location, histology, differentiation, and metastasis to other sites. Patients with bone metastasis had poor prognosis which was associated with ethnicity, tumor location, lymph node involvement, and treatment. Our findings provide important hints for tailed patient management and for further mechanistic investigations.

## Introduction

Gastric cancer, the majority of which is gastric adenocarcinoma (GaC), ranks 5th in cancer incidence and is the 4th leading cause of cancer-related mortality globally, with about 1,089,000 new cases and around 769,000 associated deaths in 2020 (1–3). Metastatic GaCs (mGaCs) with distant involvement comprise approximately 30%–44% of all GaCs at diagnosis and are associated with poor prognosis due to rapid progression (1, 4).

We previously found that among patients with mGaC, the proportions of cases with bone metastasis were 14% and 7% in the US and the Netherlands, respectively, within overall cases with mGaC, and 4% in both countries within cases with resected mGaC (4). While bone metastasis is rarer among metastatic GaCs, it can happen synchronous with or years after management of early GaCs due to residual micro-metastasis and its incidence has been rising as disease-related survival improves (5–7). Bone metastasis can destroy bone marrow and cause early onset of pathologic fracture, paralysis, hematological disorders, compromise of chemotherapy, and tremendous pain, greatly deteriorating survival and reducing quality of life (8, 9). Early detection of bone metastasis is important in preventing associated complications and guiding clinical management (10). There have been few reports on the factors associated with bone metastasis and its prognostic impact among patients with GaC, which may be due to the rarity and/or underestimation at diagnosis (11, 12).

In this comprehensive study, we used a large population-based cohort to describe characteristics of patients with GaC and bone metastasis compared to those with distant metastasis sparing bone, explore factors associated with bone metastasis in different GaC groups, calculate GaC-specific cumulative mortalities at different time points using cumulative incidence function, and reveal prognostic factors using competing risk models within GaC patients with bone metastasis. Our findings may aid to tailed management of this special patient subgroup.

## Methods

### Patients

After signing the appropriate agreement and obtaining the data use approval, independent patient-level data were retrieved from the Surveillance, Epidemiology, and End Results-18 program by the National Cancer Institute (NCI), which is an authoritative source of information on cancer statistics in the US and which collects data from population-based cancer registries. The NCI staff work with the North American Association of Central Cancer Registries (NAACCR) to guide all state registries to achieve data content and compatibility acceptable for pooling data. The program is the only comprehensive source of population-based information in the US which includes cancer stage at diagnosis and patient survival data (13).

We included patients with microscopically confirmed primary invasive adenocarcinoma of the stomach in 2010 through 2016 (**Figure S1**). The eligible time period was determined based on the availability of metastasis site information. Signet-ring cell carcinoma (SRC), a special type of adenocarcinoma, was included (3). Cases with diagnosis based on death certificate only or autopsy, with previous cancers, with ineligible histology (squamous cell carcinoma, sarcoma/gastrointestinal stromal tumor, carcinoid/neuroendocrine tumor, germ-cell tumor, and lymphoma; **Table S1**) were excluded. To enable competing risk analysis, we excluded patients with unknown causes of death. Cases with unknown distant or bone metastasis status were excluded since they did not help to address our research focus-bone metastasis.

The included registries routinely collect data on patient demographics (e.g., year of diagnosis, sex, age, and ethnicity), primary tumor site, tumor morphology (where histology and differentiation can be derived), stage at diagnosis, first course of treatment (resection, chemotherapy, and radiotherapy), cause of death, and follow-up for vital status, which were analyzed in this study. The mortality data were provided by the National Center for Health Statistics. Chemotherapy and radiotherapy were registered with low sensitivity (14). Year of diagnosis was dichotomized into 2 periods: 2010–2013 and 2014–2016. Age was categorized into 5 subgroups: <50, 50–59, 60–69, 70–79, and ≥80 years. Tumor location included 3 sites: gastric cardia, fundus/body, antrum/pylorus, and others. Causes of death included cancers and non-cancer diseases.

### Statistics

We presented categorical variables as count (percentage), and continuous data as mean ± standard deviation, median (interquartile range), and described the patient, tumor, treatment, and prognosis characteristics for GaC patients with bone metastasis and those with distant metastasis sparing bone. Survival time was calculated from diagnosis until death or last follow-up, whichever occurred first. Follow-up time was estimated using the reverse Kaplan–Meier method (15).

Associations of bone metastasis versus no bone and/or distant metastasis with year of diagnosis, sex, age, ethnicity, tumor location, SRC histology, and brain, liver, and lung metastases were quantified using the multivariable-adjusted binomial logistic regression with mutual adjustment for these variables, within metastatic and overall cancers. For tumor differentiation, adjacent structure invasion, and lymph node metastasis with missing values, the associations were assessed by additionally including these variables one by one into the above model.

We computed mortality using the cumulative incidence functions (CIFs) (16), which, unlike the standard Kaplan–Meier method, allow for estimation of the incidence of the occurrence of an event while taking competing risks from other causes of death into account for cancer-specific mortalities. Overall cumulative mortality and mortalities stratified by patient, tumor, and treatment factors at various follow-up time points (6 months and 1, 2, and 3 years) were calculated. Mortality differences between groups were evaluated using Grays’ test for equality of CIFs.

Since our study focused on incidence of mortality rather than etiology, we used the Fine–Gray subdistribution hazard function regression (16) to explore prognostic factors and compare the cumulative risks of cancer-associated mortalities across categories of individual risk and prognostic factors in all patients with GaC and bone metastasis, and calculated the corresponding hazard ratios (HR_SD_) and 95% confidence intervals (CI); the model was mutually adjusted for period of diagnosis, sex, age group, ethnicity, tumor location, SRC histology, brain, liver, and lung metastases, and resection. For tumor differentiation, adjacent structure invasion, and lymph node metastasis with missing values, the associations were evaluated by additionally including these variables one by one into the above model. Chemotherapy and radiotherapy were not included into the multivariable models considering the under-ascertainment (14), and subgroup analyses were performed for patients receiving chemotherapy or radiotherapy. The proportional hazard assumption was verified both graphically using the log–log plot and analytically using the scaled Schoenfeld residual test before performing modeling survival analyses (17). We plotted both the univariable and multivariable-adjusted survival curves for overall patients stratified by bone and distant metastasis and for patients with bone metastasis stratified by patient and tumor factors.

Analyses were performed using the R 3.5.1 software (https://cran.r-project.org), with results considered statistically significant at two-sided p < 0.05.

## Results

### Patient Characteristics

**Figure 1:**
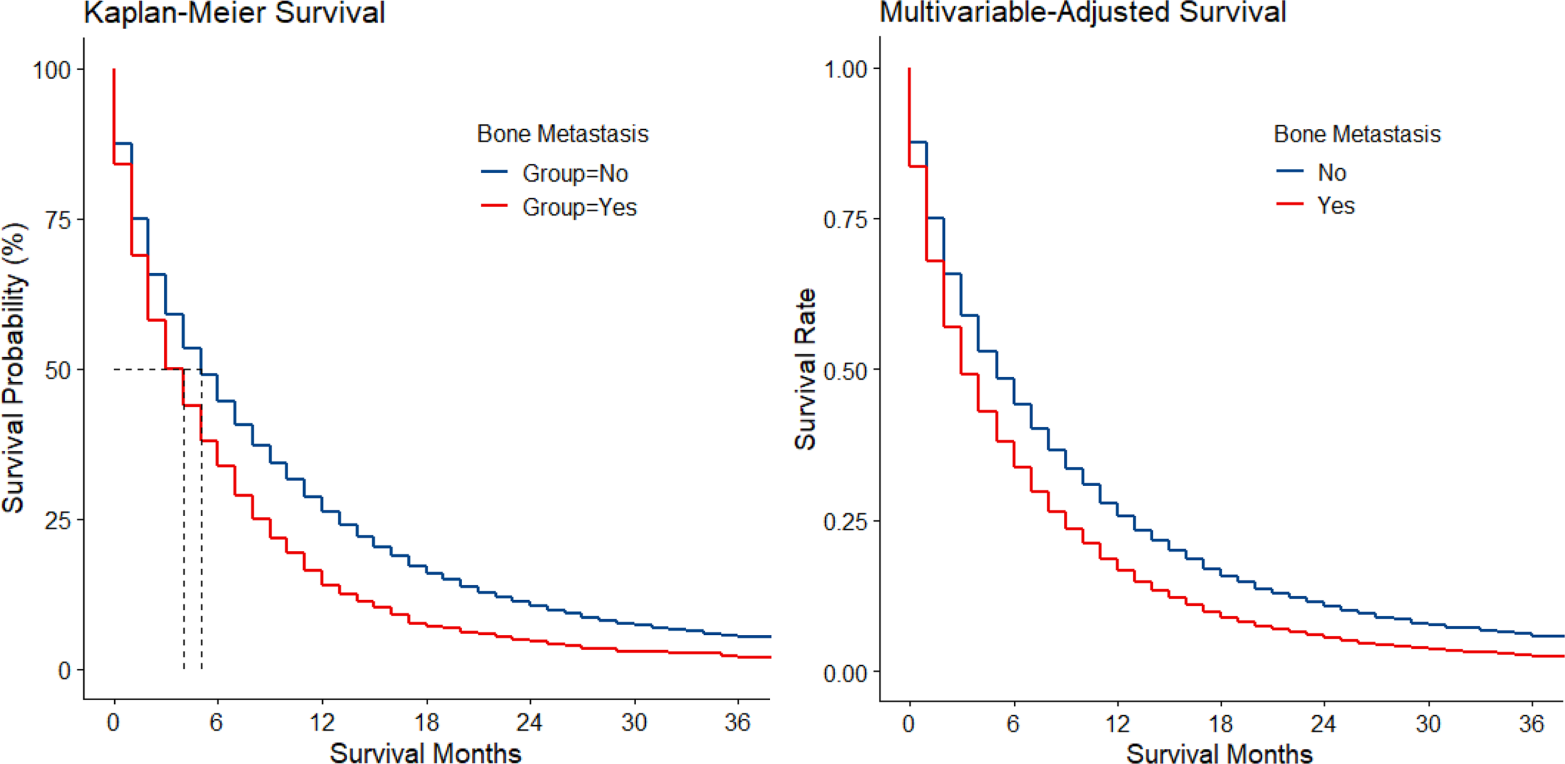
Kaplan–Meier and multivariable-adjusted survival by bone metastasis status in metastatic gastric adenocarcinoma.

Among 169,620 registered cases with gastric cancer, 11,072 patients with metastatic GaC diagnosed in 2010–2016 were included, together encompassing 6,999 person-years of follow-up; among them, 1,511 (14%) had bone metastasis (**Figure S1**). When compared to those with metastasis sparing the bone, patients with bone metastasis were slightly more often diagnosed in 2014 or later (48% vs. 45% and 43%), male (66% vs. 64% and 64%), and younger (mean age, 61 vs. 64 and 68 years) (**Table 1**). The proportion of patients <50 years was larger in those with bone metastasis (21% vs. 15% and 9%), while the proportion of people ≥80 years was smaller (8% vs. 14% and 22%). Patients with bone metastasis were more often white (74% vs. 71% and 69%) and had more often cardia cancers (62% vs. 52% and 49%) but less often antrum/pylorus cancers (19% vs. 28% and 34%). Cancers with bone metastasis were more often of SRC histology (28% vs. 21% and 18%) and poorly differentiated/undifferentiated (84% vs. 75% and 65%). Among metastatic diseases, those with bone metastasis had also more frequent metastasis to brain (5% vs. 2%) and lung (23% vs. 13%) but less often to liver (34% vs. 44%). Resection was less often performed for patients with bone metastasis (4% vs. 12% and 64%). Median survival was 4, 5, and 25 months for the 3 groups, respectively, and 11%, 16%, and 48% of patients survived at follow-up cutoff. The Kaplan–Meier and multivariable-adjusted survival curves by metastasis status are shown in **Figure 1**.

**Table 1:** Baseline characteristics of patients with metastatic gastric adenocarcinoma (with and without bone metastasis), 2010–2016^a^.

Variable	Category/comment	With bone metastasis	Without bone metastasis
**n**		1,511	9,561
**Year of diagnosis**	2014–2016	728 (48)	4,277 (45)
**Sex**	Male	1,002 (66)	6,136 (64)
**Age** (years)	As continuous	61 ± 14, 61 (52–70)	64 ± 14, 64 (54–74)
	<50	314 (21)	1,477 (15)
	50–59	354 (23)	2,108 (22)
	60–69	449 (30)	2,573 (27)
	70–79	267 (18)	2,022 (21)
	≥80	127 (8)	1,381 (14)
**Ethnicity**	White	1,120 (74)	6,817 (71)
	Black	162 (11)	1,322 (14)
	American Indian/Alaska Native	19 (1)	109 (1)
	Asian/Pacific Islander	206 (14)	1,262 (13)
	Other unspecified/unknown	4 (<1)	51 (1)
**Tumor location** *^b^*	Gastric cardia	584 (62)	3,111 (52)
	Gastric fundus/body	176 (19)	1,257 (21)
	Gastric antrum/pylorus	175 (19)	1,659 (28)
	Other	576 (38)	3,534 (37)
**Signet ring cell carcinoma**	Yes	429 (28)	2,048 (21)
**Differentiation** *^c^*	Well	13 (1)	155 (2)
	Intermediate	175 (15)	1,719 (23)
	Poor/undifferentiated	954 (84)	5,581 (75)
**Adjacent structure invasion** *^d^*	Yes	159 (23)	1,446 (26)
**Positive lymph node** *^e^*	Yes	725 (59)	4,513 (56)
**Brain metastasis**	Yes	74 (5)	147 (2)
**Liver metastasis**	Yes	511 (34)	4,194 (44)
**Lung metastasis**	Yes	347 (23)	1,283 (13)
**Resection**	Yes	55 (4)	1,115 (12)
**Chemotherapy** *^f^*	Yes	911 (60)	5,769 (60)
**Radiotherapy** *^f^*	Yes	454 (30)	1,394 (15)
**Follow-up** (months) *^g^*	As continuous	33 (18–61)	36 (16–59)
**Accumulated follow-up** (person-years)	As continuous	696	6303
**Median survival** (months)	As continuous	4 (1–8)	5 (1–13)
**Cause of death**	Alive	169 (11)	1,540 (16)
	Cancers	1,297 (86)	7,676 (80)
	Non-cancer diseases	45 (3)	345 (4)

a Categorical data are shown as count (percentage [%]), and continuous data as mean ± standard deviation, median (interquartile range). Records are complete otherwise specified below.

b The proportions of cancers located in gastric cardia, fundus/body, and antrum/pylorus were calculated within these 3 categories. Other means lesser curvature, greater curvature, overlapping lesion of stomach, and stomach (not otherwise specified).

c Missing differentiation grade: metastatic with bone metastasis, 369 (24%); metastatic without bone metastasis, 2,106 (22%).

d Missing local invasion: metastatic with bone metastasis, 828 (55%); metastatic without bone metastasis, 4,086 (43%).

e Missing positive lymph node: metastatic with bone metastasis, 290 (19%); metastatic without bone metastasis, 1,567 (16%).

f The other category for the non-surgical variables was “No/unknown,” considering the low sensitivity.

g Shown as median (interquartile range), and computed using the reverse Kaplan–Meier method.

NE, not estimable.

### Factors Associated With Bone Metastasis

In patients with metastatic GaC (**Table 2**), bone metastasis was more frequently detected in 2014 or later (OR = 1.14), in younger patients (e.g., OR_<50 vs. 60-69 years_ = 1.21, while OR_≥80 vs. 60-69 years_ = 0.56), and in SRCs (OR=1.33); it was less commonly seen in black patients (OR = 0.82), in those with gastric fundus/body (OR = 0.76) and antrum/pylorus cancers (OR = 0.61), and in those with well-differentiated (OR = 0.48) and intermediately differentiated/undifferentiated cancers (OR = 0.61). Bone metastasis was associated with more frequent brain (OR = 2.61) and lung metastases (OR = 1.93) but less often liver metastasis (OR = 0.63). Adjacent structure invasion or lymph node metastasis was not significantly associated with bone metastasis within metastatic cancers.

**Table 2:** Factors associated with bone metastasis versus no bone metastasis in patients with metastatic gastric adenocarcinoma, 2010–2016^a^.

Variable	Category	OR (95% CI)	*p*	*p_trend_*
**Year of diagnosis**	2010–2013	1.00 (reference)		
	2014–2016	1.14 (1.02–1.27)	**0.022**	
**Sex**	Male	1.00 (reference)		
	Female	0.94 (0.84–1.06)	0.324	
**Age** (years)	<50	1.21 (1.03–1.42)	**0.022**	**<0.001**
	50–59	0.97 (0.83–1.13)	0.662	
	60–69	1.00 (reference)		
	70–79	0.78 (0.66–0.92)	**0.003**	
	≥80	0.56 (0.46–0.69)	**<0.001**	
**Ethnicity**	White	1.00 (reference)		0.102
	Black	0.82 (0.69–0.99)	**0.035**	
	American Indian/Alaska Native	1.01 (0.61–1.66)	0.981	
	Asian/Pacific Islander	1.08 (0.92–1.28)	0.362	
	Other unspecified/unknown	0.51 (0.18–1.42)	0.196	
**Tumor location**	Gastric cardia	1.00 (reference)		**<0.001**
	Gastric fundus/body	0.76 (0.63–0.92)	**0.004**	
	Gastric antrum/pylorus	0.61 (0.50–0.73)	**<0.001**	
	Other *^b^*	0.87 (0.76–0.99)	**0.034**	
**Signet ring cell carcinoma**	No	1.00 (reference)		
	Yes	1.33 (1.16–1.52)	**<0.001**	
**Differentiation**	Well	0.48 (0.27–0.86)	**0.014**	**<0.001**
	Intermediate	0.61 (0.51–0.73)	**<0.001**	
	Poor/undifferentiated	1.00 (reference)		
**Adjacent structure invasion**	No	1.00 (reference)		
	Yes	0.89 (0.73–1.08)	0.233	
**Positive lymph node**	No	1.00 (reference)		
	Yes	1.08 (0.95–1.23)	0.230	
**Brain metastasis**	No	1.00 (reference)		
	Yes	2.61 (1.95–3.49)	**<0.001**	
**Liver metastasis**	No	1.00 (reference)		
	Yes	0.63 (0.55–0.71)	**<0.001**	
**Lung metastasis**	No	1.00 (reference)		
	Yes	1.93 (1.68–2.22)	**<0.001**	

a Odds ratios and 95% confidence intervals for the variables listed in the first column except tumor differentiation were calculated using the logistic regression model mutually adjusted for these variables. For tumor differentiation, adjacent structure invasion, and positive lymph node with missing values, the association was assessed by additionally including these variables one by one into the above multivariable-adjusted model. Statistically significant p values are highlighted in bold.

b Lesser curvature, greater curvature, overlapping lesion of stomach, and stomach (not otherwise specified).

OR, odds ratio; CI, confidence interval; -, not estimable.

### Overall and Stratified Survival of Patients With Bone Metastasis

**Figure 2:**
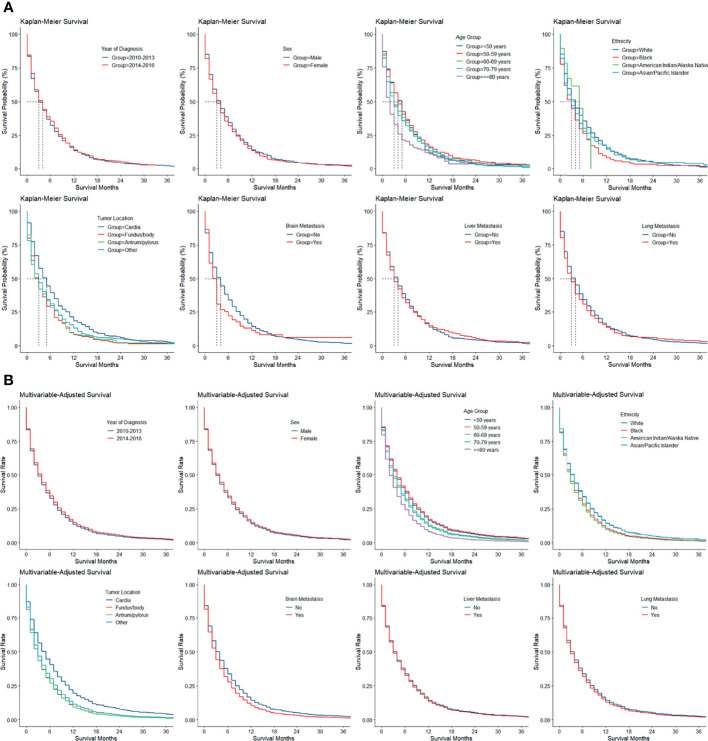
**(A)** Kaplan–Meier and **(B)** multivariable-adjusted survival by patient and tumor factors in gastric adenocarcinoma with bone metastasis.

The Kaplan–Meier and multivariable-adjusted survival of patients with bone metastasis stratified by period of diagnosis, sex, age group, ethnicity, tumor location, and brain, liver, and lung metastasis is illustrated in **Figure 2**.

Using cumulative incidence functions, overall and stratified cancer-specific mortality was calculated for patients with bone metastasis (**Table 3**). The speed of increase in mortality slowed with longer follow-up. In total patients, the 6-month mortality was already as high as 56%; the 1-year mortality was 74%, and the 2-year (83%) and 3-year mortalities (85%) were similar. Mortalities stratified by sex, age group, ethnicity, SRC histology, adjacent structure invasion, brain, liver, and lung metastasis, and radiotherapy were similar across categories, while patients with cardia cancers had lower mortalities within the 1st year of follow-up (vs. fundus/body and antrum/pylorus cancers: 6 months, 54% vs. 65% and 59%; 1 year, 74% vs. 82% and 82%), and those with poorly differentiated/undifferentiated cancers (e.g., vs. intermediately differentiated cancers: 6 months, 58% vs. 52%; 3 years, 88% vs. 84%) and lymph node metastasis (e.g., 6 months, 57% vs. 53%; 3 years, 89% vs. 82%) had higher mortalities throughout the follow-up periods. While patients who underwent resection had lower mortalities than those who did not, the difference quickly decreased with longer follow-up and became unapparent at 3 years (e.g., 6 months, 34% vs. 57%; 3 years, 82% vs. 85%). Interestingly, patients who received chemotherapy had lower mortalities than those who did not or whose receipt of chemotherapy was unknown within the first 1 year (e.g., 6 months, 46% vs. 73%; 1 year, 73% vs. 76%), while the comparison pattern reversed afterward (e.g., 2 years, 86% vs. 78%; 1 year, 89% vs. 79%).

**Table 3:** Cumulative incidences of cancer-specific mortality (%) at various follow-ups in patients with gastric adenocarcinoma and bone metastasis, overall and stratified^a^.

Group	Category	Follow-up time				*p* ^b^
		6 months	1 year	2 years	3 years	
		CIM (95% CI)	CIM (95% CI)	CIM (95% CI)	CIM (95% CI)	
**Overall**		56 (54–59)	74 (72–77)	83 (81–85)	85 (83–87)	
**Sex**	Male	57 (53–60)	75 (72–78)	84 (81–86)	87 (84–89)	0.354
	Female	56 (51–60)	73 (68–76)	81 (77–84)	82 (78–85)	
**Age** (years)	<50	52 (46–58)	75 (69–79)	82 (77–87)	84 (79–88)	0.354
	50–59	54 (49–60)	73 (68–78)	83 (78–87)	86 (82–90)	
	60–69	57 (52–61)	76 (72–80)	83 (79–86)	86 (82–89)	
	70–79	58 (52–64)	72 (66–77)	84 (79–88)	86 (81–90)	
	≥80	66 (57–74)	74 (65–81)	81 (72–87)	82 (73–88)	
**Ethnicity**	White	54 (51–57)	73 (70–76)	82 (80–85)	85 (83–87)	0.057
	Black	63 (55–70)	80 (72–85)	84 (77–89)	86 (79–91)	
	Asian/Pacific Islander	60 (53–67)	74 (67–80)	84 (77–88)	84 (77–88)	
**Tumor location**	Gastric cardia	54 (50–58)	74 (70–77)	85 (82–88)	89 (85–91)	**0.001**
	Gastric fundus/body	65 (57–72)	82 (75–87)	87 (81–92)	87 (81–92)	
	Gastric antrum/pylorus	59 (52–66)	82 (75–87)	88 (82–92)	88 (82–92)	
	Other *^b^*	55 (51–59)	70 (66–74)	77 (74–81)	80 (76–83)	
**Signet ring cell carcinoma**	No	57 (54–60)	74 (71–76)	83 (81–85)	85 (83–87)	0.932
	Yes	55 (50–60)	76 (71–80)	82 (78–86)	85 (81–88)	
**Differentiation**	Intermediate	52 (44–60)	71 (64–78)	81 (74–87)	84 (77–89)	**0.017**
	Poor/undifferentiated	58 (55–61)	76 (73–79)	85 (83–88)	88 (86–90)	
**Adjacent structure invasion**	No	54 (50–58)	75 (71–79)	87 (83–89)	89 (86–92)	0.968
	Yes	59 (51–67)	78 (70–84)	83 (76–89)	85 (78–91)	
**Positive lymph node**	No	53 (48–57)	70 (65–74)	80 (76–83)	82 (78–85)	**0.037**
	Yes	57 (53–60)	77 (73–80)	86 (83–88)	89 (86–91)	
**Brain metastasis**	No	56 (53–59)	74 (72–77)	83 (81–85)	85 (83–87)	0.656
	Yes	64 (52–74)	75 (63–83)	80 (68–88)	80 (68–88)	
**Liver metastasis**	No	56 (53–59)	75 (72–77)	83 (80–85)	85 (83–88)	0.852
	Yes	57 (52–61)	73 (69–77)	83 (79–86)	84 (81–88)	
**Lung metastasis**	No	57 (54–59)	75 (73–78)	84 (82–87)	87 (85–89)	0.078
	Yes	55 (50–60)	71 (65–75)	77 (72–82)	79 (74–83)	
**Resection**	No	57 (55–60)	75 (73–77)	83 (81–85)	85 (83–87)	**0.029**
	Yes	34 (21–46)	57 (42–69)	70 (55–81)	82 (66–91)	
**Chemotherapy**	No/unknown	73 (69–76)	76 (73–80)	78 (74–81)	79 (76–82)	**0.001**
	Yes	46 (42–49)	73 (70–76)	86 (83–88)	89 (86–91)	
**Radiotherapy**	No/unknown	57 (54–60)	74 (71–77)	83 (80–85)	84 (82–87)	0.337
	Yes	55 (50–59)	75 (70–79)	84 (80–87)	87 (83–90)	

a Calculated using the cumulative incidence function. Results for subgroups with case number <50 are not shown. Statistically significant p values are highlighted in bold.

b Using Gray’s test for equality of cumulative incidence functions.

CIM, cumulative incidence of mortality; CI, confidence interval.

### Prognostic Factors in Patients With GaC and Bone Metastasis Who Received Chemotherapy

Since data on chemotherapy and radiotherapy were registered with low sensitivity albeit high specificity (14), and considering the fact that chemotherapy is the standard of care for patients with metastatic GaC regardless of bone metastasis, we focused on patients with GaC and bone metastasis who received chemotherapy. Using the Fine–Gray subdistribution hazard regression model, factors associated with cancer-specific mortality in cases with GaC and bone metastasis who received chemotherapy are shown in **Table 4**. American Indians/Alaskan Natives had higher mortalities than white patients (HR_SD_ = 1.72). Compared to gastric cardia cancers, gastric antrum/pylorus cancers were associated with a higher mortality risk (HR_SD_ = 1.29). Lymph node metastasis increased the mortality risk by 18% unit. Cases undergoing resection had 35% unit mortality risk reduction.

**Table 4:** Fine–Gray subdistribution hazard ratios for cancer-specific mortality among patients with gastric adenocarcinoma and bone metastasis who were treated with chemotherapy^a^.

Variable	Category	HR (95% CI)	*p*	*p_trend_*
**Period of diagnosis**	2010–2013	1.00 (reference)		
	2014–2016	0.88 (0.76–1.01)	0.072	
**Sex**	Male	1.00 (reference)		
	Female	0.94 (0.80–1.11)	0.478	
**Age group**	< 50 years	0.97 (0.79–1.18)	0.746	0.877
	50–59 years	0.93 (0.77–1.12)	0.420	
	60–69 years	1.00 (reference)		
	70–79 years	0.98 (0.79–1.22)	0.867	
	≥ 80 years	1.10 (0.76–1.61)	0.610	
**Ethnicity**	White	1.00 (reference)		**0.015**
	Black	1.18 (0.92–1.50)	0.187	
	American Indian/Alaska Native	1.72 (1.22–2.41)	**0.002**	
	Asian/Pacific Islander	0.97 (0.77–1.23)	0.808	
	Other unspecified/unknown	0.29 (0.03–3.37)	0.326	
**Tumor location**	Gastric cardia	1.00 (reference)		**0.048**
	Gastric fundus/body	1.21 (0.93–1.57)	0.154	
	Gastric antrum/pylorus	1.29 (1.02–1.63)	**0.035**	
	Other *^b^*	0.96 (0.81–1.14)	0.659	
**Signet ring cell carcinoma**	No	1.00 (reference)		
	Yes	0.98 (0.82–1.17)	0.824	
**Differentiation**	Well	0.94 (0.37–2.42)	0.905	0.480
	Intermediate	0.87 (0.70–1.09)	0.226	
	Poor/undifferentiated	1.00 (reference)		
**Adjacent structure invasion**	No	1.00 (reference)		
	Yes	1.03 (0.79–1.35)	0.831	
**Positive lymph node**	No	1.00 (reference)		
	Yes	1.18 (1.01–1.39)	**0.044**	
**Brain metastasis**	No	1.00 (reference)		
	Yes	1.23 (0.82–1.84)	0.326	
**Liver metastasis**	No	1.00 (reference)		
	Yes	1.04 (0.89–1.21)	0.641	
**Lung metastasis**	No	1.00 (reference)		
	Yes	1.04 (0.87–1.24)	0.685	
**Resection**	No	1.00 (reference)		
	Yes	0.65 (0.46-0.92)	**0.014**	

a Hazard ratios and 95% confidence intervals for the variables listed in the first column except tumor differentiation were calculated using the Fine–Gray subdistribution hazard model mutually adjusted for these variables. For tumor differentiation, adjacent structure invasion, and positive lymph node with missing values, the association was assessed by additionally including these variables one by one into the above multivariable-adjusted model. Statistically significant p values are highlighted in bold.

b Lesser curvature, greater curvature, overlapping lesion of stomach, and stomach (not otherwise specified).

HR, hazard ratio; CI, confidence interval; NE, not estimable due to small case number.

## Discussion

In this large population-based cohort study, we analyzed more than 1,500 cases with bone metastasis among more than 11,000 GaC patients encompassing approximately 7,000 person-years of follow-up and comprehensively described their characteristics and compared them to those with distant metastasis sparing bone. We identified unique risk features specific for bone metastasis which were further validated using multivariable analyses. Taking competing risks into account, we further showed the overall and stratified cancer-specific mortalities of GaC patients with bone metastasis using CIFs, representing a more accurate estimation of prognosis. Comparisons of cumulative mortalities were further supported by multivariable competing risk analysis. Our findings offer important clues for screening and management of bone metastasis in GaCs.

Cases with bone metastasis took up 5% and 14% of overall and metastatic cases with GaC, respectively. While rarer compared to metastasis from other sites like breast and lung, GaC bone metastasis is rapidly progressing and can mark impaired survival and reduce quality of life. The overall cumulative mortality increased greatly from 56% at 6 months to 85% at 3 years, with a median survival time of only 4 months. With the advancement of diagnosis techniques and management, bone metastasis was more frequently diagnosed and had better outcome in more recent periods.

There are several ways to detect GaC bone metastasis. It can be diagnosed using bone scintigraphy and PET/CT; PET/CT may be more effective with greater specificity in the initial staging workup, and conversely, bone scanning and PET/CT may be similarly effective for the detection of metachronous bone metastasis (18–20). Specific antibodies can help with the diagnosis (6). Bone alkaline phosphatase may be a surrogate marker of bone metastasis in GaC patients (8). High-circulating tumor cell concentrations may be a biological hallmark of GaC with bone metastasis at diagnosis and can be used as an early, direct, and definitive indicator of therapeutic response, superior to imaging analysis which may typically take several months to complete (21, 22). Microangiopathic hemolytic anemia and leukoerythroblastic blood film may herald bone-metastatic GaC (23). Quantification of CD44v6 mRNA can suggest micro-metastasis in the bone marrow (24). Despite these methods, it remains difficult to early identify GaC bone metastasis, which can be frequently misdiagnosed and underestimated.

Notably, 12 patients with bone metastasis in our study cohort did not show primary site lesion (T0 disease), and 239 had disease only confined to mucosa or submucosa (T1 cancer). GaC bone metastasis partly depends on angiogenesis with the involvement of infiltrating mast cells positive for tryptase (25, 26), and this process may be RANKL-independent (27). Bone marrow-derived myofibroblasts can promote GaC growth and metastasis through the activation of the TGF-β1 and IL-6/STAT3 signaling pathways (28). Nevertheless, the driving force of bone metastasis remains largely unknown (29). It is important to explore bone metastasis-associated risk factors to facilitate efficient identification of this clinically significant situation.

We found that bone metastasis in GaC appeared more frequently with later diagnosis period, younger age, SRC histology, non-black ethnicities, cardiac location, and poorer differentiation. Later diagnosis period and cardiac cancers were associated with lower mortality hazards, while American Indians/Alaskan Natives, poorer differentiation, and lymph node metastasis with increased mortality risks. Previous studies also show that SRC, poorer differentiation, and lymph node involvement are associated with more frequent bone metastasis (30, 31). In patients with GaC and bone metastasis, smoking history, poorer performance status, poorer differentiation, and higher levels of LDH, CEA, and CA 19-9 were associated with poorer prognosis, while chemotherapy and zoledronic acid with prolonged survival (32, 33). Extraosseous metastasis was reported to be associated with poorer survival in GaC patients with bone metastasis (34).

Notably, patients <50 years took up 21% of all cases with bone-metastatic GaC compared to 8% for those ≥80 years. Older patients generally had slower cancer progression, and the preference of bone metastasis for younger people with longer expected survival is worth the clinician’s attention. Cardiac cancers differ from non-cardiac tumors in many aspects and are associated with poorer survival (35), and we found them to be associated with more often bone metastasis. Interestingly, they were associated with better survival in GaC patients with bone metastasis, especially within the 1st year of follow-up. SRC is a special histology type and may be more biologically aggressive and less sensitive to chemotherapy, making immediate upfront resection of vital importance. Poorer differentiation indicates greater heteromorphism, conferring malignant cells with greater metastasis potential, and was found to be associated with inferior survival. Lymph node metastasis may be an intermediate transitional status before distant metastasis occurs. While it was associated with more frequent distant metastasis, 41% of cases with bone-metastatic GaCs had negative lymph nodes. Node metastasis rather than adjacent structure invasion was correlated with survival in bone-metastatic GaCs. GaCs with multiple metastatic sites including bone are clinically noteworthy, while brain, liver, or lung metastasis was not significantly associated with worse prognosis in our study.

We found that 4% of patients with GaC and bone metastasis underwent resection and that the possible protective effects of treatment (resection and chemotherapy) appeared most prominent within the 1st year after diagnosis, which may highlight the need of continued or maintenance therapy for medically fit patients with metastatic disease. Notably, while resection was associated with higher survival in patients with GaC and bone metastasis, causality and definitive treatment benefits could not be derived from our observational study. Currently, there is no treatment guideline for GaC with bone metastasis, and palliative chemotherapy and bisphosphonates (e.g., zoledronic acid) have been the main pharmacological treatment option, which can enhance survival and improve quality of life (36–39). Tailored treatment is vital (40). A case report described a 53-year-old male GaC patient with bone metastasis who underwent total gastrectomy and palliative metastasectomy and who received combination chemotherapy with S-1 and cisplatin and survived for about 5 years after surgery with markedly improved quality of life (41). Antigen peptide-pulsed dendritic cell-activated cytotoxic T lymphocyte immunotherapy may effectively help to reduce bone metastasis burden in mGaC (42). Exploration of ideal management pathways for this special patient group is warranted.

It may be difficult to identify bone metastasis early in patients with GaC. The reasons may include the following: first, in patients with GaC bone metastasis is much rarer compared to other major distant metastases (e.g., liver, lung, and peritoneal metastases); second, bone metastasis is a “deep-seated” lesion, and not all patients may have typical or obvious symptoms at the early stage of bone metastasis, which may be associated with the detailed pathological changes. Third, while bone scan remains the modality of choice to screen bone metastases, the diagnostic value of bone scan in detecting bone metastases limited to specific locations in cancer patients has a moderately high false-negative diagnostic rate (43).

Our findings may offer important clues for the identification of bone metastases in patients with GaC and may help with the management and tailoring of the treatment for patients with GaC and metastatic bone involvement. For example, special attention may need to be paid to younger patients with GaC, patients with gastric cardia cancers, patients with signet-ring cell carcinomas, and those with poorly differentiated/undifferentiated cancers, who should be carefully screened for bone metastasis, since they were shown to be prone to having more often bone metastasis. For patients with brain and/or lung metastasis, bone metastasis may also need to be carefully screened for. The overall and stratified cumulative incidences of cancer-specific mortality at various follow-ups in patients with GaC and bone metastasis could help with clinical counseling; mortalities estimated using the cumulative incidence function took into account competing risks and could be more valid than the Kaplan–Meier estimates, which could be overestimations of mortalities in the presence of competing risks (16, 44). Considering the very poor outcome of patients with GaC and bone metastasis and the quickly increasing cumulative mortalities due to cancer with a high mortality rate of 56% already at 6 months, cancer-directed therapies (chemotherapy and/or others) may need to be initiated at an early stage for selected otherwise fit patients with GaC involving the bone, in addition to best supportive care. Notably, for patients with gastric antrum/pylorus cancers and those with positive lymph nodes who have high mortality risks even if they receive chemotherapy, the benefits and harms which such treatment could bring should be carefully weighed, and a decision on management may need to be made after comprehensively taking the patients’ preference, life expectancy, quality of life, performance and nutrition statuses, medical costs, and various other factors into account beyond the limited prolongation of life which aggressive management may lead to.

Our observational study shared some common limitations with other population-based registry-based studies. Data on some other factors possibly associated with bone metastasis (e.g., environmental and genetic risk factors) and prognostic factors (e.g., comorbidities and health conditions) were unavailable. Nevertheless, we had included most of the common risk and prognostic factors in multivariable modelling. Some variables (e.g., tumor local invasion and lymph node involvement) had missing values for metastatic cases; we did not initially include them in multivariable analyses but added them separately into multivariable models to evaluate the associations with them. In this population-based study, chemotherapy and radiotherapy were registered with low sensitivity albeit high specificity (14), and detailed information on treatment (e.g., type, regimens, agents, and course) was unavailable. Accordingly, we did not include them into multivariable survival analyses and performed subgroup analyses of cases receiving chemotherapy or radiotherapy. Furthermore, the findings were based on US patients and may not be generalizable to other nations especially Asians, where gastric cancer is far more prevalent. Analyses of datasets from other countries are strongly encouraged.

To our knowledge, this is the largest population-based real-world study using individual-level data and robust statistics to address bone metastasis in patients with GaC. The careful and strict case enrollment, use of comprehensive multivariable competing risk analytical methods, and meticulous subgroup analyses enable this report to provide robust, valid, and useful references for individualized bone-metastatic GaC management.

## Conclusions

In GaC patients, bone metastasis was associated with various clinicopathologic factors including age, ethnicity, tumor location, histology, differentiation, and metastasis to other sites. Patients with bone metastasis had poor prognosis which was associated with ethnicity, tumor location, lymph node involvement, and treatment. Our findings provide clinically important and useful hints and evidence for tailed management of patients with bone-metastatic GaC and for further mechanistic investigations.

## Data Availability Statement

Publicly available datasets were analyzed in this study after signing the appropriate agreement and obtaining the data use approval. These data can be found as follows: Surveillance, Epidemiology, and End Results Program Research Data (www.seer.cancer.gov).

## Ethics Statement

Ethical review and approval were not required for this study using the Surveillance, Epidemiology, and End Results Program database after signing the appropriate agreement and obtaining the data use approval in accordance with the local legislation and institutional requirements. Written informed consent for participation was not required for this study in accordance with the national legislation and the institutional requirements.

## Author Contributions

Conception or design: LH, WH, and JZ. Acquisition, analysis, or interpretation of data: LH, YZ, YS, WH, and JZ. Drafting of the manuscript: LH. Critical revision of the manuscript for important intellectual content: YZ, YS, WH, and JZ. Statistical analysis: LH. Administrative, technical, or material support: WH and JZ. All authors contributed to the article and approved the submitted version.

## Funding

This study was supported by the Fund for Medical Center on Aging (GB202103) and Start-up Fund for the Introduction of High Level Talents by Ruijin Hospital, Shanghai Jiao Tong University School of Medicine. The funder had no involvement in the study design; in the collection, analysis, or interpretation of data; in the writing of the report; or in the decision to submit the paper for publication.

## Conflict of Interest

The authors declare that the research was conducted in the absence of any commercial or financial relationships that could be construed as a potential conflict of interest.

## Publisher’s Note

All claims expressed in this article are solely those of the authors and do not necessarily represent those of their affiliated organizations, or those of the publisher, the editors and the reviewers. Any product that may be evaluated in this article, or claim that may be made by its manufacturer, is not guaranteed or endorsed by the publisher.
