# Supplementary material for: Bone Metastasis From Gastric Adenocarcinoma—What Are the Risk Factors and Associated Survival? A Large Comprehensive Population-Based Cohort Study
Source: Front Oncol. 2022 Mar 25;12:743873. doi: 10.3389/fonc.2022.743873 (PMC8989732; doi:10.3389/fonc.2022.743873)
Supplement: Supplementary file 1 [file DataSheet_1.docx]

**Supplementary Materials**

**Supplementary Results**

**Table S1.** Inclusion and exclusion codes according to International Classification of Diseases for Oncology, Third Edition

**Figure S1.** Case selection flow diagram

**Table S1.** Inclusion and exclusion codes according to International Classification of Diseases for Oncology, Third Edition

| **Category** |  | **Code** |
| --- | --- | --- |
| **Topology** | Inclusion | C16, C16.0, C16.1, C16.2, C16.3, C16.4, C16.5, C16.6, C16.8, C16.9 |
|  | Exclusion | - |
| **Morphology** | Inclusion | 8000-8089, 8140-8389, 8440-8579 |
|  | Exclusion | 8013, 8052, 8053, 8060, 8070-8078, 8081, 8083, 8084, 8152, 8153, 8156, 8160, 8170, 8240-8243, 8246, 8249, 8252, 8390, 8590, 8680, 8700, 8711, 8720, 8800-8805, 8810, 8811, 8830, 8840, 8850-8852, 8858, 8890, 8891, 8895-8897, 8900, 8902, 8910, 8912, 8920, 8930, 8931, 8935, 8936, 8960, 9040, 9041, 9064, 9071, 9080, 9090, 9100, 9120, 9364, 9380, 9490, 9500, 9540, 9560, 9580 |
| **Behavior** | Inclusion | 3 |
|  | Exclusion | 0, 2 |

**
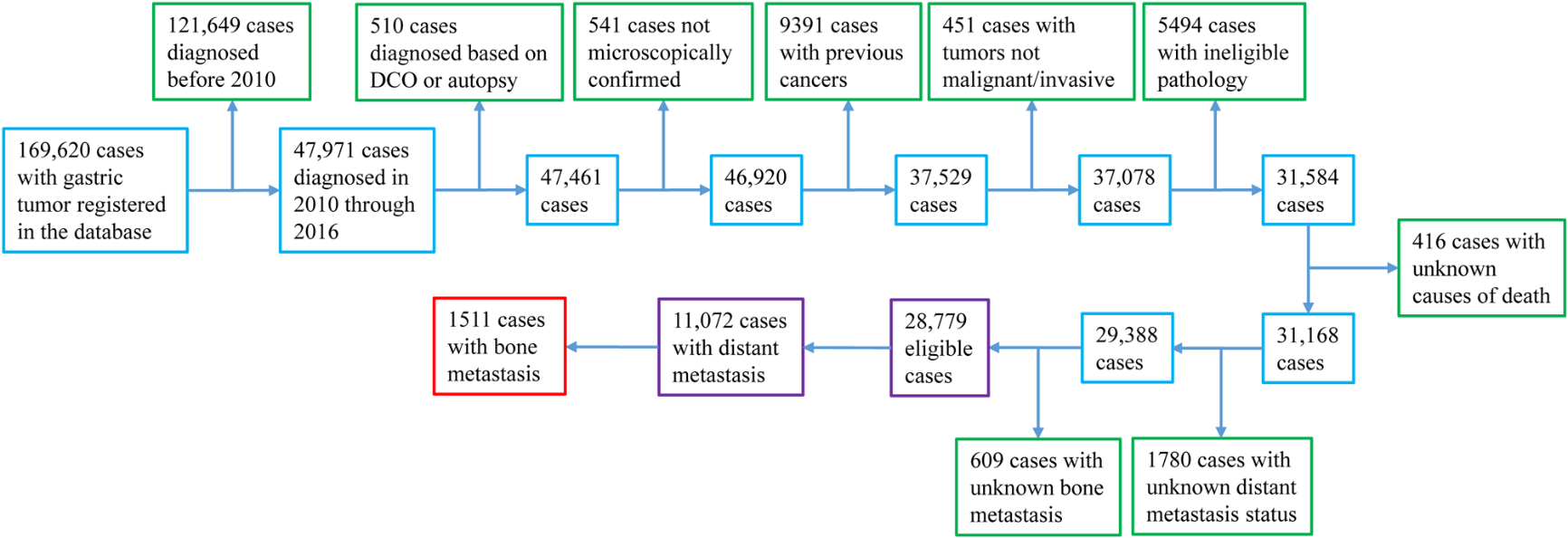
**

**Figure S1.** Case selection flow diagram
